# Supplementary material for: Segmentation metric misinterpretations in bioimage analysis
Source: Nat Methods. 2023 Jul 27;21(2):213–6. doi: 10.1038/s41592-023-01942-8 (PMC10864175; doi:10.1038/s41592-023-01942-8)
Supplement: Supplementary file 1 — Supplementary Table 1. [file 41592_2023_1942_MOESM1_ESM.pdf]

nature methods

Brief Communication https://doi.org/10.1038/s41592-023-01942-8

Segmentation metric misinterpretations in bioimage analysis

In the format provided by the authors and unedited

Supplementary Information

**Formula**	$\frac{TP}{TP+FP}$	$\frac{TP}{TP+FN}$	$2\frac{P \cdot R}{P+R} = \frac{2TP}{2TP+FP+FN}$	$\frac{TP}{TP+FP+FN}$
**Pixel-based name**	Precision	Recall	Dice score	Intersection over union (IoU)
**Object-based name**	Precision	Recall	F1 score	Threat score

Supplementary Table 1. Simple metrics (top row) that can be defined either on a pixel level (middle row) or object level (bottom row).
