# Supplementary material for: Segmentation metric misinterpretations in bioimage analysis
Source: Nat Methods. 2023 Jul 27;21(2):213–6. doi: 10.1038/s41592-023-01942-8 (PMC10864175; doi:10.1038/s41592-023-01942-8)
Supplement: Supplementary file 2 — Reporting Summary. [file 41592_2023_1942_MOESM2_ESM.pdf]

nature portfolio

Corresponding author(s): Peter Horvath

Last updated by author(s): May 21, 2023

Reporting Summary

Nature Portfolio wishes to improve the reproducibility of the work that we publish. This form provides structure for consistency and transparency in reporting. For further information on Nature Portfolio policies, see our Editorial Policies and the Editorial Policy Checklist.

Statistics

For all statistical analyses, confirm that the following items are present in the figure legend, table legend, main text, or Methods section.

n/a	Confirmed	
☐	☒	The exact sample size (*n*) for each experimental group/condition, given as a discrete number and unit of measurement
☒	☐	A statement on whether measurements were taken from distinct samples or whether the same sample was measured repeatedly
☒	☐	The statistical test(s) used AND whether they are one- or two-sided<br>*Only common tests should be described solely by name; describe more complex techniques in the Methods section.*
☒	☐	A description of all covariates tested
☒	☐	A description of any assumptions or corrections, such as tests of normality and adjustment for multiple comparisons
☐	☒	A full description of the statistical parameters including central tendency (e.g. means) or other basic estimates (e.g. regression coefficient) AND variation (e.g. standard deviation) or associated estimates of uncertainty (e.g. confidence intervals)
☒	☐	For null hypothesis testing, the test statistic (e.g. *F*, *t*, *r*) with confidence intervals, effect sizes, degrees of freedom and *P* value noted<br>*Give P values as exact values whenever suitable.*
☒	☐	For Bayesian analysis, information on the choice of priors and Markov chain Monte Carlo settings
☒	☐	For hierarchical and complex designs, identification of the appropriate level for tests and full reporting of outcomes
☒	☐	Estimates of effect sizes (e.g. Cohen's *d*, Pearson's *r*), indicating how they were calculated

Our web collection on statistics for biologists contains articles on many of the points above.

Software and code

Policy information about availability of computer code

Data collection	No software was used for data collection.
Data analysis	We used some of the evaluation code provided by StarDist (https://github.com/stardist/stardist, version 0.8.3). All of the source code used to process the submissions and create the ranking correlations can be found at https://bitbucket.org/biomag/metric-code/

For manuscripts utilizing custom algorithms or software that are central to the research but not yet described in published literature, software must be made available to editors and reviewers. We strongly encourage code deposition in a community repository (e.g. GitHub). See the Nature Portfolio guidelines for submitting code & software for further information.

Data

Policy information about availability of data

All manuscripts must include a data availability statement. This statement should provide the following information, where applicable:

- Accession codes, unique identifiers, or web links for publicly available datasets
- A description of any restrictions on data availability
- For clinical datasets or third party data, please ensure that the statement adheres to our policy

Images and corresponding ground truth masks are publicly available for the DSB2018 and Sartorius Challenges. As for the MIDOG2021 challenge, the final stage evaluation data is private and thus not available. Submission files from the competitors are available upon request.
DSB2018 data: https://www.kaggle.com/competitions/data-science-bowl-2018/data

Sartorius data: https://www.kaggle.com/competitions/sartorius-cell-instance-segmentation/data
MIDOG2021 data: https://imig.science/midog2021/download-dataset/

Human research participants

Policy information about studies involving human research participants and Sex and Gender in Research.

Reporting on sex and gender	Our study did not involve human research participants.
Population characteristics	Our study did not involve human research participants.
Recruitment	Our study did not involve human research participants.
Ethics oversight	Our study did not involve human research participants.

Note that full information on the approval of the study protocol must also be provided in the manuscript.

Field-specific reporting

Please select the one below that is the best fit for your research. If you are not sure, read the appropriate sections before making your selection.

☒ Life sciences ☐ Behavioural & social sciences ☐ Ecological, evolutionary & environmental sciences

For a reference copy of the document with all sections, see nature.com/documents/nr-reporting-summary-flat.pdf

Life sciences study design

All studies must disclose on these points even when the disclosure is negative.

Sample size	We provide sample sizes from 3 different competitions:<br>The DSB2018 competition included a total of 2263 submissions for the second stage evaluation,<br>the Sartorius challenge included 1304 submissions for the final stage evaluation,<br>the MIDOG2021 challenge included 13 submissions for the final stage evaluation.<br>We included every submission available from the competitions for our analyses, thus, these sample sizes were sufficient for the experiments.
Data exclusions	No data was excluded from the analyses.
Replication	Running the evaluation scripts on the submission data will reproduce the data of the study.
Randomization	Not relevant to study, because we included all of the submissions from 3 different competitions, calculating the correlation between the participants' placements according to different metrics does not require any randomization of the samples/submissions.
Blinding	No group allocation was done, thus blinding was also not relevant to study.

Reporting for specific materials, systems and methods

We require information from authors about some types of materials, experimental systems and methods used in many studies. Here, indicate whether each material, system or method listed is relevant to your study. If you are not sure if a list item applies to your research, read the appropriate section before selecting a response.

Materials & experimental systems

n/a	Involved in the study	
☒	☐	Antibodies
☒	☐	Eukaryotic cell lines
☒	☐	Palaeontology and archaeology
☒	☐	Animals and other organisms
☒	☐	Clinical data
☒	☐	Dual use research of concern

Methods

n/a	Involved in the study	
☒	☐	ChIP-seq
☒	☐	Flow cytometry
☒	☐	MRI-based neuroimaging
